# A novel bifunctional N-acetylglutamate synthase-kinase from *Xanthomonas campestris *that is closely related to mammalian N-acetylglutamate synthase

**DOI:** 10.1186/1471-2091-8-4

**Published:** 2007-04-10

**Authors:** Qiuhao Qu, Hiroki Morizono, Dashuang Shi, Mendel Tuchman, Ljubica Caldovic

**Affiliations:** 1 Children's Research Institute, Children's National Medical Center, The George Washington University, 111 Michigan Avenue NW, Washington DC 20010, USA; 2 Division of Neurosciences, Beckman Research Institute City of Hope National Medical Center, 1450 E Duarte Road, Duarte CA 91010, USA

## Abstract

**Background:**

In microorganisms and plants, the first two reactions of arginine biosynthesis are catalyzed by N-acetylglutamate synthase (NAGS) and N-acetylglutamate kinase (NAGK). In mammals, NAGS produces an essential activator of carbamylphosphate synthetase I, the first enzyme of the urea cycle, and no functional NAGK homolog has been found. Unlike the other urea cycle enzymes, whose bacterial counterparts could be readily identified by their sequence conservation with arginine biosynthetic enzymes, mammalian NAGS gene was very divergent, making it the last urea cycle gene to be discovered. Limited sequence similarity between *E. coli *NAGS and fungal NAGK suggests that bacterial and eukaryotic NAGS, and fungal NAGK arose from the fusion of genes encoding an ancestral NAGK (*argB*) and an acetyltransferase. However, mammalian NAGS no longer retains any NAGK catalytic activity.

**Results:**

We identified a novel bifunctional N-acetylglutamate synthase and kinase (NAGS-K) in the Xanthomonadales order of gamma-proteobacteria that appears to resemble this postulated primordial fusion protein. Phylogenetic analysis indicated that xanthomonad NAGS-K is more closely related to mammalian NAGS than to other bacterial NAGS. We cloned the NAGS-K gene from *Xanthomonas campestis*, and characterized the recombinant NAGS-K protein. Mammalian NAGS and its bacterial homolog have similar affinities for substrates acetyl coenzyme A and glutamate as well as for their allosteric regulator arginine.

**Conclusion:**

The close phylogenetic relationship and similar biochemical properties of xanthomonad NAGS-K and mammalian NAGS suggest that we have identified a close relative to the bacterial antecedent of mammalian NAGS and that the enzyme from *X. campestris *could become a good model for mammalian NAGS in structural, biochemical and biophysical studies.

## Background

**Figure 1:**
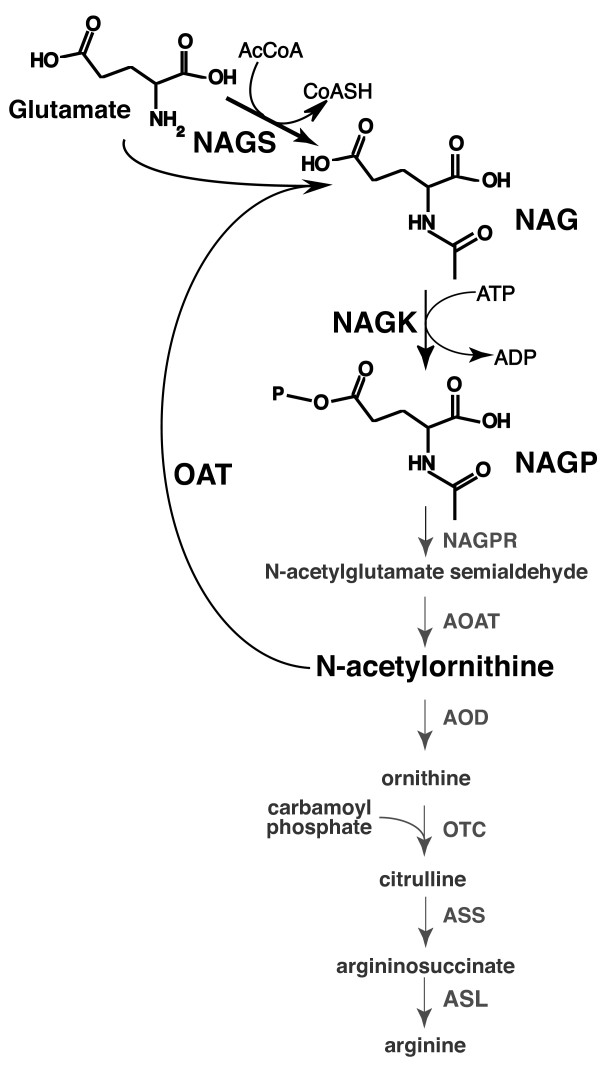
**Arginine biosynthesis in microorganisms and plants**. Most microbes and plants utilize a "cyclical" pathway in which ornithine acetyltransferase recycles the acetyl group from acetylornithine to glutamate. In these organisms, NAGS replenishes NAG lost in cell division and growth. Some bacteria, such as *E. coli *and *Xanthomonadales*, utilize a "linear" pathway in which NAGS catalyzes formation of the first intermediate of arginine biosynthesis. Abbreviations: NAGP, N-acetylglutamyl phosphate; NAGPR, N-acetylglutamyl phosphate reductase; AOAT, acetylornithine aminotransferase; OAT, ornithine acetyltransferase; AOD, acetylornithine deacetylase, OTC, ornithine transcarbamylase, ASS, argininosuccinate synthase; ASL, argininosuccinate lyase.

**Figure 2:**
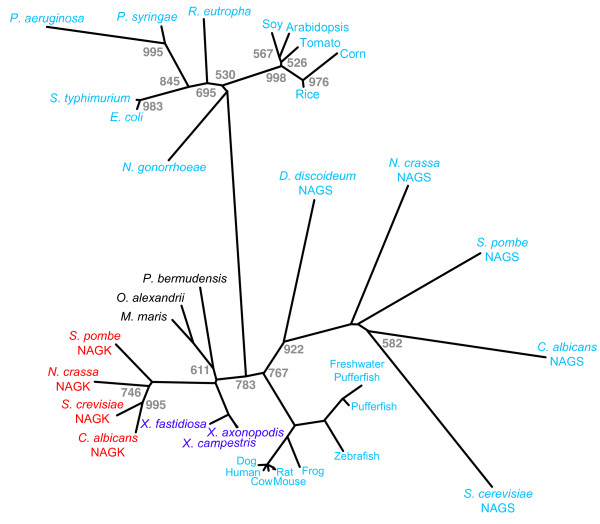
**Phylogenetic tree of NAGS protein sequences from bacteria, fungi, algae, plants and vertebrates, and fungal NAGK**. The tree was generated by the neighbor joining method using Phylip 3.6. Numbers indicate bootstrap values from 1000 replicas. Bootstrap values equal to 1000 are not indicated in the figure due to spatial constraints. NAGS, NAGSK and NAGS/K proteins are shown in blue, red and purple typeface, respectively. Proteins from *M. maris*, *O. alexandrii *and *P. bermudensis *are shown in black typeface because their ability to catalyze synthase reaction, kinase reaction, or both has not been examined experimentally. NAGS, NAGK and NAGS-K from the following organisms were included in the phylogenetic analysis: human – *Homo sapiens*, mouse – *Mus musculus*, rat – *Ratus norvegicus*, cow – *Bos torus*, dog – *Canis familiaris*, frog – *Xenopus tropicalis*, zebrafish – *Danio rerio*, pufferfish – *Fugu rubripes*, freshwater pufferfish – *Tetraodon nigroviridis*, soy – *Glycine max*, corn – *Zea mayis*, tomato – *Solanum lycopersicum*, rice – *Oryza sativa*, Arabidopsis – *Arabidopsis thaliana*, *S. cerevisiae *– *Saccharomyces cerevisiae*, *S. pombe *– *Schizosaccharomyces pombe*, *C. albicans *– *Candida albicans*, *N. crassa *– *Neurospora crassa*, *D. discoideum *– *Dictiostelium discoideum*, *X. campestris *– *Xanthomonas campestris*, *X. axonopodis *– *Xanthomonas axonopodis*, *X. fastidiosa *– *Xylella fastidiosa*, *M. maris *– *Maricaulis maris*, *O. alexandrii *– *Oceanicaulis alexandrii*, *P. bermudensis *– *Parvulalcula bermudensis*, *P. aeruginosa *– *Pseudomonas aeruginosa*, *P. syringiae *– *Pseudomonas syringiae*, *N. gonorrhoeae *– *Neisseria gonorrhoeae*, *S. typhimurium *– *Salmonella typhimurioum*, *E. coli *– *Escherichia coli*.

The biosynthesis of arginine in microorganisms and plants is accomplished in eight enzymatic steps (Figure 1, [1,2]). The first reaction in arginine biosynthesis is N-acetylation of glutamate by N-acetylglutamate synthase (NAGS; EC 2.3.1.1). The second reaction of arginine biosynthesis is phosphorylation of the γ-carboxyl group of NAG by N-acetylglutamate kinase (NAGK; EC 2.7.2.8) to produce N-acetylglutamylphosphate (NAGP) which is subsequently converted to ornithine in two more steps (Figure 1, [1]). In fungi, plants and many bacteria, a cyclic pathway occurs in the first portion of arginine biosynthesis (Figure 1, [1,2]). Organisms with a cyclic pathway use ornithine acetyltransferase (EC 2.3.1.35) to regenerate N-acetylglutamate (NAG) via transfer of acetyl group from N-acetylornithine to glutamate. The role of NAGS in organisms with ornithine acetyltransferase is to replenish the NAG that is lost due to cell growth and division [2]. In these microorganisms, both NAGS and NAGK are inhibited by arginine [2] whereas in organisms with the linear pathway, NAGS is the target of feedback inhibition by arginine. Enterobacteria, *Vibrio*-like bacteria, a delta-proteobacterium *Myxococcus xanthus*, *Xanthomonas campestris *and the archaeon *Sulfolobus solfataricus *do not posses ornithine acetyltransferase; these organisms have a linear pathway, where NAGS is the sole source of NAG and therefore essential for the arginine biosynthesis [1,3-9]. In bacteria, two broad classes of NAGS have been found: "full-length" sequences of approximately 440 amino acids that resemble *Echerichia coli *NAGS, and "short" NAGS such as those in *Mycobacterium tuberculosis *of approximately 170 amino acids in length [8,10,11]. In both the linear and cyclic arginine biosynthetic pathways, the same enzymes and intermediates are used in the final four steps from ornithine to arginine (Figure 1). In eukaryotes, arginine biosynthesis is partitioned across organelles with fungal NAGS and NAGK located in the mitochondria [12-19] and the cognate plant enzymes located in the chloroplasts [20].

In mammals, amphibians, fish and some invertebrates, NAGS, carbamylphosphate synthase, together with the last four enzymes of the arginine biosynthesis pathway and arginase comprise the urea cycle, a metabolic pathway that converts waste nitrogen in the form of ammonia into urea [2,21]. Urea functions as an osmolyte in some fish, whereas its role in teleost fish is still unclear [22]. In mammals and other land animals, the urea cycle protects the central nervous system from the toxic effects of ammonia [2,21]. In these organisms, NAGS is localized in the mitochondria and catalyzes formation of NAG, which is an essential allosteric activator of carbamylphosphate synthase I (CPSI) [21]. Interestingly, the enzymatic activity of mammalian NAGS is not inhibited but rather increases in the presence of arginine [23-26]. The physiological significance of NAGS activation by arginine is not clear since its estimated intramitochondrial concentration appears to be sufficiently high to saturate NAGS and result in its maximal activity [24,27,28].

Unlike other enzymes of the urea cycle, which are conserved across phyla [29-31], the similarity between mammalian and fungal NAGS is relatively low [32] and the fungal and bacterial enzymes are even more divergent [12]. Closer examination of the *Saccharomyces cerevisiae *NAGS and NAGK primary sequences shows the C-terminal regions of both enzymes share similarity with the C-terminal half of *E. coli *NAGS, while the N-terminal region of *S. cerevisiae *NAGK has homology to the *E. coli *NAGK [33]. The N-terminal region of *S. cerevisiae *NAGS appears more divergent and whether it has any catalytic function is unknown [12,33]. Limited sequence similarity between NAGK from *Neurospora crassa *and NAGS from *E. coli *led to the hypothesis that bacterial NAGS and fungal NAGK may have common ancestry [14]. The mammalian NAGS also shares similarity with fungal NAGK [32], enough that the gene was misannotated as a NAGK in initial releases of the human genome project.

An evolutionary model was proposed in a recent analysis of the arginine biosynthesis pathway that the present day NAGS arose by fusion of ancestral bacterial *argB *(NAGK) gene and an acetyltransferase gene [8]. This suggests that NAGS and fungal NAGK evolved from a gene encoding a bifunctional enzyme with both N-acetylglutamate synthase and kinase activities.

In our search for homologs of mammalian NAGS, we identified a novel gene in *Xanthomonas campestris *that encodes a bifunctional protein which can catalyze the first two reactions of the arginine biosynthesis pathway. We used phylogenetic analysis to show that this novel N-acetylglutamate synthase – N-acetylglutamate kinase (NAGS-K or *argA-B*) is more closely related to mammalian NAGS than to other bacterial NAGS (*argA*) genes. In this paper, we report on the cloning and characterization of this novel *argA-B *gene and its XcNAGS-K protein.

## Results

### Identification of the *argA-B *gene coding for a bifunctional NAGS-K

Genes coding for the bifunctional NAGS-K proteins were identified in the genomes of several Xanthomonadales based on the similarity of their amino acid sequence to human and mouse NAGS genes. These genes were annotated as *argB *in *X. campestris*, *Xanthomonas axonopodis *and *Xylella fastidiosa*; proteins encoded by these *argB *genes are 80–98% identical and 85–98% similar to each other but they also showed 34–36% identity and 41–43% similarity to human and mouse NAGS. The similarity between the putative NAGS gene from Xanthomonadales and mammalian NAGS genes spans what we term the "conserved segment" of NAGS [34]. These xanthomonad putative NAGS genes represent the first bacterial genes with significant sequence similarity to mammalian NAGS. To confirm this, we performed phylogenetic analysis of known and candidate NAGS and NAGK protein sequences. Included in the analysis were sequences of enzymatically validated NAGS [4,15,32,34-38] as well as predicted proteins that showed similarity to human NAGS and *E. coli argA *in BLAST searches of GenBank. The NAGS from plants, beta-proteobacteria, gamma-proteobacteria including *argB *gene products from Xanthomonadales and three marine alpha-proteobacteria all share similarity with *E. coli *NAGS. Sequences with higher similarity to human NAGS were those from mammals, other vertebrates, fungi and amoeba, but also the fungal NAGK. Interestingly, the *argB *gene products from Xanthomonadales and three marine alpha-proteobacteria were more similar to human NAGS than even the fungal NAGS. In archaeal genomes there were no candidate NAGS genes with sequences similar either to full-length NAGS from *E. coli *or mammalian NAGS; genes that encode shorter proteins have been annotated as NAGS in archaea [8,10].

We assembled NAGS protein sequences from 31 organisms including both bacteria and eukaryotes for phylogenetic analysis. Included were enzymatically validated as well as candidate NAGS from mammals (human [34], mouse [32], rat, cow and dog), candidate NAGS from other vertebrates (frog, zebrafish, pufferfish and freshwater pufferfish), two characterized fungal NAGS from *S. cerevisiae *and *N. crassa *[15,36,37] and two candidate fungal NAGS from *Schisosaccharomyces pombe *and *Candida albicans*, a candidate NAGS from an amoeba *Dictyostelium discoideum*, candidate NAGS enzymes from plants (soy, tomato, corn, rice and arabidopsis), bacterial NAGS from *E. coli*, *Salmonella typhimurium*, *Pseudomonas aeruginosa *and *Pseudomonas syringae *that have been previously characterized [4,35,36,38], candidate NAGS from *Ralstonia eutropha *and *Neisseria gonorrhoeae *(beta-proteobacteria), three putative NAGS from Xanthomonadales (*X. campestris*, *X. axonopodis *and *X. fastidiosa*) and three candidate NAGS from marine alpha-proteobacteria (*Maricaulis maris*, *Oceanicaulis alexandrii *and *Parvularcula bermudensis*). The putative NAGS from the bacterium *Ralstonia eutropha *was included in this analysis because it had a high similarity score to both NAGS from *E. coli *and humans (E-values: 7 × 10^-108 ^and 1 × 10^-5^, respectively). Fungal NAGK from *S. cerevisiae *[13], *N. crassa *[14], *Schizosaccharomyces pombe *[18] and *C. albicans *[16] were included based on the previously noted similarity between *N. crassa *NAGK and *E. coli *NAGS [14].

Figure 2 shows a phylogenetic tree generated using the neighbor joining method with a PMB amino acid substitution matrix [39]. Phylogenetic trees were also generated using the neighbor joining with a Jones-Taylor-Thornton amino acid substitution matrix and parsimony methods [40]. Both phylogenetic trees had the same topology as the one shown in Figure 2. NAGS and NAGK sequences clustered in two groups. NAGS sequences from vertebrates, Xanthomonadales, three marine alpha-proteobacteria, fungi and amoeba, and fungal NAGK are in one cluster indicating that they are more closely related to each other than to the members of another cluster containing plant and other bacterial NAGS. These two groupings of NAGS and NAGK were strongly supported by high bootstrap values (>700) in all of the phylogenetic trees that were generated.

**Figure 3:**
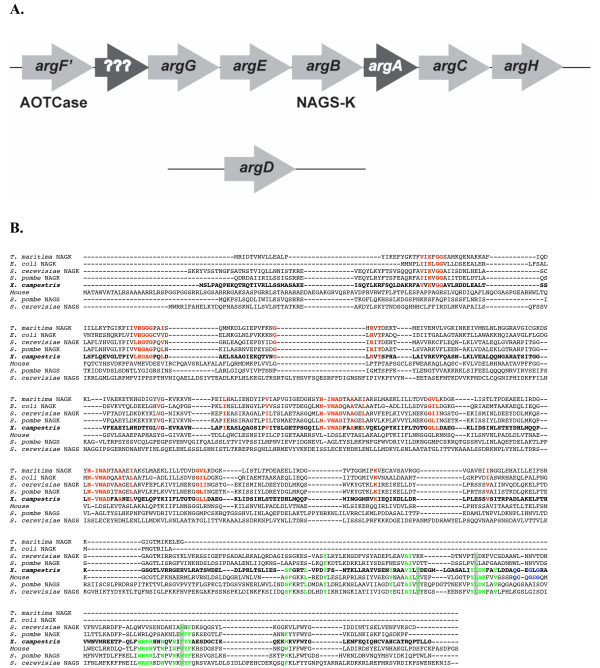
**Organization of the arginine operon in *X. campestris *and amino acid sequence conservation between NAGS-K and both NAGS and NAGK**. **A**. The arginine operon in *X. campestris*. *ArgC*, *argD*, *argG *and *argH *genes were identified based on their similarity with homologs in other bacteria. *ArgF' *encodes acetylornithine transcarbamylase (AOTCase) and *argE *gene product can catalyze deacetylation of acetylornithine as well as acetylcitrulline [7]. The *argA *gene was annotated based on its similarity with other acetyltransferases. Genes labeled with question marks encode hypothetical proteins of unknown function. ArgD gene is located elsewhere in the genome and not in the same cluster as other eight genes. **B**. Alignment of NAGS from mouse, *S. cerevisiae *and *S. pombe*, NAGS-K from *X. campestris*, and NAGK from *T. maritima*, *E. coli*, *S. cerevisiae *and *S. pombe*. Residues that are important for catalysis of NAGK are shown in red. Residues that are conserved in vertebrate and fungal NAGS are shown in green. Residues that are mutated in patients with NAGS deficiency are highlighted in grey. Conserved residues that are presumed to be part of AcCoA binding site are shown in blue.

The high similarity and close evolutionary relationship between vertebrate NAGS and products of genes annotated as *argB *in Xanthomonadales led to the hypothesis that the xanthomonad genes encoded NAGS and not NAGK. However, closer inspection of the arginine operon from *X. campestris *(Figure 3A) revealed a gene coding for a hypothetical protein of 203 amino acids in length annotated as *argA*, in addition to the *argB *gene that we had identified as a close relative of vertebrate NAGS. No other genes in the operon or elsewhere in the *X. campestris *genome were annotated as, or similar to, *argB *or NAGK. Eight genes are clustered together in the arginine operon of *X. campestris*; *argD* gene is located elsewhere in the genome (Figure 3A). Arginine biosynthesis in Xanthomonadales is unusual because instead of ornithine transcarbamylase (*argF*) they use acetylornithine transcarbamylase (AOTCase or *argF'*) for biosynthesis of acetylcitrulline, which is subsequently deacetylated by the *argE *gene product [7]. Query of the NCBI Conserved Domain database with the sequence of the hypothetical ArgAprotein from *X. campestris *revealed that it is similar to acetyltransferases of the GNAT superfamily. We cloned the hypothetical *argA *gene from the *X. campestris*, overexpressed it in *E. coli*, and purified the protein. Under conditions where the *X. campestris *ArgBprotein showed abundant NAGS activity (enzymatic acetylation of glutamate), the ArgA protein had none (data not shown). It is likely that the ArgA recombinant protein was inactive because it lacked an important cofactor or interacting partner or it does not use acetyl coenzyme A (AcCoA) and/or glutamate as substrates.

The sequence of the *argB *gene product from *X. campestris *was aligned with mouse NAGS, NAGK with known three-dimensional structures from *E. coli *and *T. maritima *(Figure 3B) [41,42], and fungal NAGS and NAGK from *S. cerevisiae *and *S. pombe*. Amino acids that are important for binding of the NAGK substrates, ATP and NAG, as well as those involved in catalysis of NAGP formation [41] were conserved in the *argB* gene product from *X. campestris*, bacterial and fungal NAGK. These amino acids, which are also conserved in other bacterial *argB *genes [41], are highlighted in red in Figure 3B. This figure also shows the conservation of amino acids (in green), found in the C-terminal half of NAGS from vertebrates and fungi [43] with those of the xanthomonad *argB *gene product. Three of these conserved amino acids, highlighted in gray, are mutated in patients with NAGS deficiency [43,44] and therefore are important for the function of mammalian NAGS. Moreover, the amino acid motif (R/Q)XXGXG (shown in blue in Figure 3B), characteristic of the AcCoA binding sites [45,46] is present in the mammalian NAGS and the xanthomonad *argB *gene product.

Based on this evidence we hypothesized that genes annotated as *argB *in Xanthomonadales encoded a dual-function enzyme that catalyzed the first two reactions of the arginine biosynthesis: formation of NAG from glutamate and AcCoA, and phosphorylation of NAG to form NAGP [1]. We henceforth refer to these genes as *argA-B *and their products NAGS-K. *The argA-B *designation reflects the two reactions catalyzed by the product of this gene: the *argA *reaction (synthesis of NAG) followed by the *argB *reaction (phosphorylation of NAG), which are catalyzed by two discrete proteins, ArgAand ArgBin *E. coli*.

### Cloning of the *argA-B *gene from *X. campestris *and purification of recombinant NAGS-K

**Figure 4:**
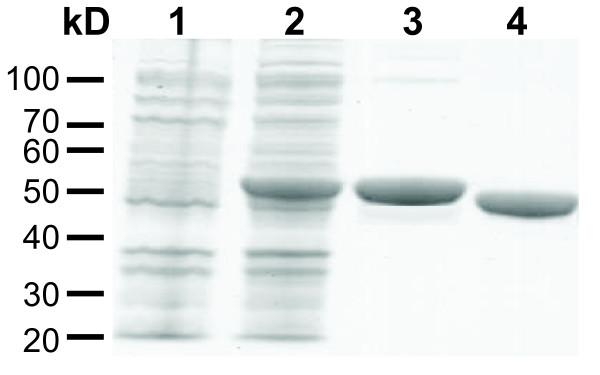
**Purification of recombinant XcNAGS-K**. The XcNAGS-K with the N-terminal polyhistidine tag was overexpressed in *E. coli *and purified using nickel-affinity column. Lane 1 – cell lysate before induction of XcNAGS-K overexpression; lane 2 – cell lysate; lane 3 – purified XcNAGS-K with the polyhistidine tag; lane 4 – purified XcNAGS-K after removal of the tag.

The *argA-B *gene was cloned from *X. campestris *genomic DNA and inserted into an *E. coli *expression plasmid to produce recombinant protein. We were able to overexpress the XcNAGS-K protein and purify it to homogeneity (Figure 4). The denatured protein migrated as a single 50 kDa band, in good agreement with the predicted molecular weight of 50,149 Da. The purified protein was tested for its ability to catalyze the formation of NAG and NAGP and was biochemically characterized.

Table 1 shows that purified enzyme can catalyze the formation of NAG from glutamate and AcCoA (synthase activity) as well as the formation of NAGP from NAG and ATP (kinase activity), confirming that it is bifunctional. The synthase activity was completely inhibited in the presence of 1 mM L-arginine suggesting that this enzyme is likely a target of feedback inhibition by the final product of arginine biosynthesis. The kinase activity of XcNAGS-K was only slightly inhibited by 1 mM L-arginine (Table 1). When ATP or NAG were omitted from the reaction, or when EDTA was added, kinase activity was absent (data not shown). We also tested the substrate specificity of XcNAGS-K, and Table 2 shows that L-glutamate was the only suitable acetyl acceptor in the synthase half-reaction and that CTP and UTP could not replace ATP as substrates for the kinase half-reaction. We were unable to test if XcNAGS-K could catalyze the coupled reaction for the formation of NAGP from glutamate, AcCoA and ATP, because AcCoA reacted with hydroxylamine and FeCl_3_, and produced a colored compound even in control reactions where heat inactivated enzyme was added.

**Table 1:** Initial measurements of the N-acetylglutamate synthase and kinase activities of the *X. campestris *enzyme. One unit of activity is defined as one μmole of product produced in one minute.

	**Synthase Activity** (units/mg of protein)		**Kinase Activity ** (units/mg of protein)	
		1 mM Arginine		1 mM Arginine
Purified Enzyme	76.04 ± 3.36^a^	1.55 ± 0.07	1.95 ± 0.04	1.55 ± 0.07
Boiled Enzyme	nd^b^	nd	nd	nd

^a^specific activity measurements were done in triplicate ^b^not detectable

**Table 2:** Substrate specificity of XcNAGS-K. The concentrations of L-glutamate and its alternatives were 50 mM. The concentrations of ATP, CTP and UTP were 20 mM. One unit of activity is defined as one μmole of product produced in one minute.

**Substrate**	**Synthase Activity **(units/mg of protein)	**Kinase Activity **(units/mg of protein)
L-Glutamate	31.5 ± 0.0^a^	-
D-Glutamate	nd^b^	-
L-Glutamine	nd	-
L-Aspartate	nd	-
ATP	-^c^	4.84 ± 0.00
CTP	-	0.11 ± 0.01
UTP	-	0.07 ± 0.01

^a^specific activity is an average of two measurements ^b^not detectable ^c^not assayed

### Biochemical properties of XcNAGS-K

**Figure 5:**
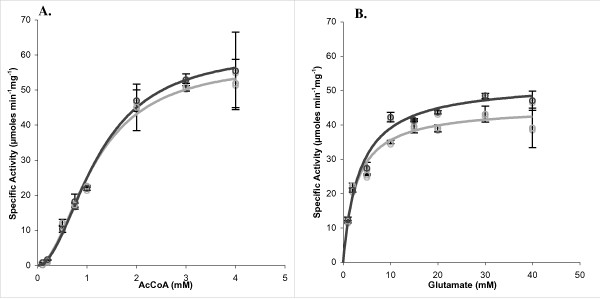
**Dependence of the synthase reaction of XcNAGS-K on the concentration of AcCoA and glutamate**. **A**. AcCoA concentration was varied between 0 and 4 mM while glutamate concentration was fixed at 50 mM. **B**. Glutamate concentration was varied between 0 and 50 mM while AcCoA was fixed at 2 mM. The assays were performed either with XcNAGS-K fused with a polyhistidine tag (black) or without affinity tag (gray).

K_m_-values for AcCoA and L-glutamate and the corresponding maximal velocities were measured for purified XcNAGS-K. We also examined whether the presence of a polyhistidine affinity tag alters the biochemical properties of the enzyme. Figure 5A illustrates that the dependence of the rate of NAG formation on the concentration of AcCoA deviates from Michaelis-Menten behavior and is sigmoidal. This suggests cooperativity with respect to binding of AcCoA. The presence of a polyhistidine tag did not significantly alter the apparent K_m_-values for the substrates (K_m_^app^), maximal velocities (V_max_) or Hill coefficients (h) (Table 3). Figure 5B shows the dependence of the rate of NAG formation on the concentration of L-glutamate. N-terminal polyhistidine tag did not significantly alter K_m_^app ^and V_max _for L-glutamate (Table 3).

**Table 3:** Biochemical properties of the XcNAGS-K with respect to its synthase activity.

		**XcNAGS-K**^a^	**H_6_XcNAGS-K**^b^
**AcCoA**	**V**_max _^e ^(μmoles min^-1 ^mg^-1^)	**58.1 ± 6.2**^c^	**61.6 ± 7.6**
	**K**_m_^app ^(mM)	**1.3 ± 0.4**	**1.5 ± 0.5**
	**h**	**1.9 ± 0.4**	**2.0 ± 0.5**
**Glu**	**V**_max _^e ^(μmoles min^-1 ^mg^-1^)	**45.4 ± 1.8**^d^	**52.8 ± 1.2**
	**K**_m_^app ^(mM)	**2.8 ± 0.5**	**3.5 ± 0.4**

^a^XcNAGS-K without affinity tag ^b^XcNAGS-K with the polyhistidine tag and thrombin protease recognition sequence ^c^values for AcCoA represent fitting parameters to Hill equation and the associated errors ^d^values for glutamate represent fitting parameters to Michaelis-Menten equation and the associated errors ^e^the V_max_-values apply to infinite concentrations of glutamate and AcCoA only, not all substrates

We were not able to examine the K_m_-values and turnover numbers for NAG and ATP because of the limited sensitivity of the kinase colorimetric assay [47]. The rate of NAGP formation was proportional to enzyme concentration and linear with time under the conditions used to measure activity (see Additional file 1), but the dynamic range of the kinase colorimetric assay limited the usable enzyme concentrations and reaction times needed to perform detailed kinetic analysis.

### Regulation of XcNAGS-K by arginine

**Figure 6:**
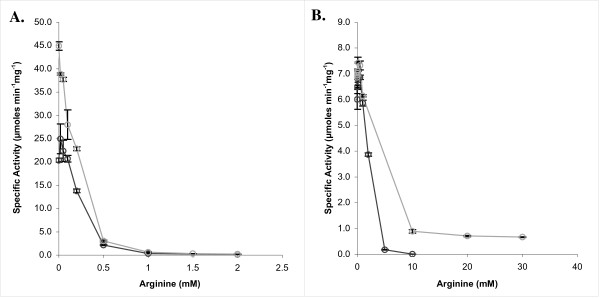
**Effects of arginine on enzymatic activities of XcNAGS-K**. Synthase (A) and kinase (B) activities of the XcNAGS-K with the polyhistidine tag (black) or without tag (gray) were measured in the presence of increasing arginine concentrations. Arginine concentration was varied between 0 and 2 mM in the synthase assay (A) and between 0 and 30 mM in the kinase assay (B).

In microorganisms and plants, NAGS is subject to feedback inhibition by L-arginine [2]. NAGK from *E. coli*, which uses the linear arginine biosynthesis pathway, is not inhibited by arginine, although NAGK in bacterial fungi and plants that use the cyclic pathway are negatively regulated by arginine [2]. Arginine has the opposite effect in human, mouse and rat NAGS, where it increases activity [23-26]. Both the synthase and kinase activities of XcNAGS-K are inhibited by arginine (Figure 6). However, the synthase activity appears to be approximately 25 times more sensitive to arginine than kinase activity; in XcNAGS-K, without an affinity-tag, the half-maximal synthase and kinase activities were observed at 0.2 and 5.1 mM of arginine, respectively. The presence of an affinity tag did not affect the inhibition of the synthase activity of XcNAGS-K (Figure 6A). However, the arginine concentration sufficient for half-inhibition of the kinase activities of XcNAGS-K with and without polyhistidine differed two-fold (Figure 6B). Inhibition of both reactions by arginine is consistent with XcNAGS-K being a target for feedback inhibition in arginine biosynthesis.

### Effect of pH on the enzymatic activities of XcNAGS-K

The effect of pH on XcNAGS-K activity was examined using purified protein with an intact polyhistidine affinity tag. Synthase and kinase activities were measured in a series of buffers with different pH values. Table 4 shows that the pH optimum for the synthase is 9.0 while it is 6.0 for the kinase. To rule out that buffering compounds used to adjust the pH may have affected the enzymatic activities, we tested whether NaCl, MES, MOPS, Tris-HCl or sodium acetate themselves had an effect on either synthase or kinase activities. The effects of variations in ionic strength of the differing buffering compounds were examined by adding 200 mM NaCl to assay reactions. Table 5 illustrates that the addition of 100 mM NaCl, MOPS and MES did not significantly alter synthase activity, while 100 mM sodium acetate and 200 mM NaCl inhibited the activity by about 22%. The kinase activity of XcNAGS-K did not change appreciably when 100 mM NaCl, 100 mM MOPS, 100 mM Tris-HCl and 200 mM NaCl were added to the reaction mixture. The difference in optimal pH for the two reactions is probably due to different requirements for interactions among charged groups upon binding of substrates and catalysis.

**Table 4:** Dependence of the synthase and kinase activities on pH. Enzymatic activity is defined as the number of μmoles of product formed in one minute.

**pH**	**Synthase Specific Activity**	**pH**	**Kinase Specific Activity**
6.5	0.59 ± 0.01^a^	4.5	3.46 ± 0.42^b^
7.0	2.75 ± 0.02	5.0	4.77 ± 0.56
7.5	3.66 ± 0.10	5.5	6.15 ± 0.04
8.0	4.67 ± 0.64	6.0	7.02 ± 0.02
8.5	12.18 ± 1.42	7.0	6.59 ± 0.11
9.0	18.33 ± 1.94	7.5	6.55 ± 0.33
10.0	15.16 ± 1.73	8.0	3.99 ± 0.21

^a^numbers represent averages of two measurements and associated standard errors ^b^numbers represent averages of three measurements and associated standard errors

**Table 5:** Effects of buffer components on enzymatic activities of XcNAGS-K. Synthase activity was assayed in 100 mM Tris-HCl, pH 9.0 that contains 100 mM NaCl and compounds listed in the table. Kinase activity was assayed in 100 mM MES buffer, pH 6.0 that contained 100 mM NaCl and compounds listed in the table. Enzymatic activity is defined as the number of μmoles of product formed in one minute.

**Added Compound**	**Synthase Specific Activity **(units/mg)	**Added Compound**	**Kinase Specific Activity **(units/mg)
100 mM NaCl	41.35 ± 1.05^a^	100 mM NaCl	4.34 ± 0.15
100 mM MOPS	41.29 ± 1.55	100 mM MOPS	4.70 ± 0.11
100 mM Acetate	26.99 ± 0.64	100 mM Acetate	4.91 ± 0.05
100 mM MES	38.83 ± 0.54	100 Tris-HCl	4.07 ± 0.09
200 mM NaCl	29.91 ± 0.23	200 mM NaCl	4.75 ± 0.01

^a^numbers represent averages of three measurements and associated standard errors

## Discussion

Herein we describe a novel bifunctional N-acetylglutamate synthase and kinase (NAGS-K) from *X. campestris *which is close bacterial homolog to mammalian NAGS. In addition to sequence similarity, mammalian NAGS and *X. campestris *NAGS-K have similar biochemical properties. The affinities for AcCoA and glutamate are similar, as is the concentration of arginine needed for half-inhibition or activation of the synthase activity of XcNAGS-K and mammalian NAGS, respectively [24]. The kinase activity of the XcNAGS-K was also inhibited by arginine. However, NAG phosphorylation was approximately 25 times less sensitive to arginine than NAG synthesis activity. This difference in sensitivity to arginine could be due in part because the synthase and kinase activities are assayed separately, while *in vivo*, both activities would be inhibited concurrently since the biosynthesis of NAGP would stop as soon as the kinase is deprived of its substrate NAG.

### Functional domains of NAGS-K

**Figure 7:**
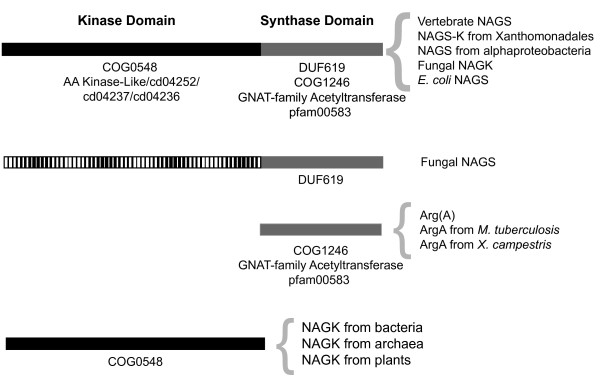
**Domain organization of NAGS and NAGK from bacteria and eukaryotes**. Kinase domain is shown in solid black. Synthase domain is shown in solid gray. The N-terminal domain of fungal NAGS is shown as a hatched rectangle. Enzyme types (NAGS or NAGK or NAGS-K) and their origins are indicated to the right of each domain architecture. Names of the conserved domains are indicated below each domain. Multiple names are used for designation of some conserved domains because of the redundancy of the conserved domain database. They are separated by slash-marks.

NAGS-K from Xanthomonadales is composed of two functional domains: the N-terminal kinase domain that belongs to the COG0548 conserved domain family and spans approximately 265 amino acids, and the DUF619 domain in the C-termini, which is approximately 160 amino acids long (Figure 7). This domain organization is found in other bacterial NAGS, vertebrate NAGS and fungal NAGK [8,10]. NAGK from archaea, plants and most bacteria are approximately 265 amino acids long and have only one COG0548 conserved domain (Figure 7). Examination of the domain relationships in the conserved domain database revealed that DUF619 is related to the COG1246 acetyltransferase domain family that is found in the C-termini of bacterial and plant NAGS proteins. The COG1246 conserved domain is also present in NAGS from *M. tuberculosis*, which is 174 amino acids long [11], and the *arg(A) *segment of the *argH(A) *gene from the two Moritella species [8,9], suggesting that a functional glutamate N-acetyltransferase could be fully contained within the 156 amino acids at the C-terminus of XcNAGS-K. Examination of the domain relationships in the conserved domain database revealed that DUF619 and COG1246 conserved domains are also related to the domain pfam00583 of the GCN5-related N-acetyltransferases (GNAT) superfamily, which is present in the putative *argA *gene in the arginine operon of *X. campestris*. This suggests that there may be two distinct active sites in the XcNAGS-K: one in the C-terminal domain that carries out the acetyltransferase function (synthase) and one in the N-terminal kinase domain, which is responsible for phosphorylation of NAG. Figure 7 shows that NAGS from fungi also have two domains: the C-terminal acetyltransferase domain that belongs to the DUF619 family and the N-terminal domain whose sequence appears to have diverged from the kinase domain present in other two-domain NAGS and fungal NAGK [8,12,33]. However, our phylogenetic analysis (Figure 2) and sequence alignment (Figure 3B) strongly suggests that fungal NAGS is related to vertebrate NAGS and fungal NAGK. It is likely that divergence of fungal NAGS sequences reflects possible protein-protein interactions [12,33] or other adaptations unique to fungi. Determination of the three-dimensional structure of the XcNAGS-K and a more detailed examination of its kinetic properties should reveal the identities and location of active site residues and lead to better understanding of how the acetyltransferase and kinase domains interact.

### Evolution of NAGS

We examined the distribution of NAGS and NAGK across the three domains of life. Although NAGK is found in archaea, eubacteria and eukaryotes, such as plants, algae and fungi, initially, we were only able to find sequences similar to either *E. coli *or mammalian NAGS in beta-proteobacteria, gamma-proteobacteria and three species of alpha-proteobacteria [48-50]. The three alpha-proteobacteria, *M. maris*, *O. alexandrii *and *P. bermudensis*, also appear to harbor acetylornithine transcarbamylase (*argF'*) genes suggesting that their arginine biosynthesis pathway is similar to the one in *X. campestris *[7]. Identification of the alpha-proteobacterial NAGS genes that are closely related to the corresponding vertebrate, fungal and algal genes and to fungal NAGK is intriguing because mitochondria are thought to have arisen by endosymbiosis between proto-eukaryotic cell and an alpha-proteobacteria. Current thought suggests that the alpha-proteobacteria of the order Rickettsiales are the extant relatives of the endosymbiont that gave rise to mitochondria, and these do not appear to have a bifunctional NAGS-K. *M. maris *and *O. alexandrii *belong to the order of Rhodobacterales and *P. bermudensis *is a member of the order Parvuralculares. Detailed analysis of the relationships among the alpha proteobacteria orders with respect to NAGS will be needed to better illuminate if the ancestry of the vertebrate NAGS has origins in the alpha proteobacteria. It is likely that, as more genomic data and candidate NAGS sequences become available, it will be possible to reconstruct the evolutionary history of NAGS in a way similar to that done for CPS, OTC and arginase [29-31].

Based on the close relationship between XcNAGS-K, other xanthomonad homologs, vertebrate NAGS and fungal NAGK, it is likely that XcNAGS-K and its homologs are extant representatives of an ancestral gene that arose by fusion of the ancestral *argB *gene encoding NAGK, and an ancestral gene encoding an acetyltransferase [8,10]. We speculate that the primordial bifunctional protein underwent gene duplication followed by divergence to evolve into the present day mammalian NAGS and fungal NAGK. The present day mammalian NAGS retained acetyltransferase enzymatic activity and lost the kinase activity whereas the present day fungal NAGK retained kinase activity and lost acetyltransferase activity. XcNAGS-K retains conservation of amino acids important for both activities (Figure 3B). The evolutionary descendant of the unfused form of the acetyltransferase, if it still exists, remains to be identified.

Many archaea and eubacteria, including *X. campestris *[GenBank:AAM41523] and *M. tuberculosis *[GenBank:NP_217263], contain genes that are between 150 and 210 amino acids long and are annotated as *argA *or NAGS. Moreover, the *argA *gene product of *M. tuberculosis *appears to have NAGS enzymatic activity [11], suggesting that the C-terminal domain of XcNAGS-K alone could be sufficient for catalysis of NAG formation. NAGS function is also contained within a 173 amino acids long arg(A) segment of the argH(A) fusion gene found in the *Moritella abyssi *and *Moritella profunda *[9,51]. All these "short" *argA *gene products belong to the COG1246 family of acetyltransferases that are distinct from the DUF619 family (Figure 7). Based on the above observation and the relationship between COG1246 and DUF619 protein families, we cannot exclude the possibility of two separate gene fusions occurred and gave rise to two families of NAGS proteins: one fusion of NAGK and acetyltransferase from the DUF619 family could have given rise to the Xanthomonad NAGS-K, NAGS from the alpha-proteobacteria *M. maris*, *O. alexandrii *and *P. bermudensis*, vertebrate NAGS, and fungal NAGS and NAGK, and a second fusion between NAGK and an acetyltransferase from the COG1246 family might have resulted in the extant plant, beta- and gamma-proteobacterial NAGS. However, data presented in Figure 2 could also be explained by horizontal gene transfer of NAGS-K from eukaryotes into Xanthomonadales, Rhodobacterales and/or Parvuralculares. Sequencing and analysis of additional microbial genomes will help to resolve which one of these evolutionary models is more likely.

The high level of divergence of NAGS compared to other genes in the arginine biosynthesis pathway could be explained by the dual role of NAGS among species (arginine biosynthesis vs. ureagenesis). In organisms with the linear arginine biosynthesis pathway and those harboring a urea cycle, NAGS plays an essential role of either catalyzing the formation of the first intermediate in the arginine biosynthesis or as a cofactor for the CPSI and CPSIII [2]. This would imply stronger selection pressure for conservation of amino acid sequence and protein function. We speculate that in microbes and plants that have the cyclical arginine biosynthesis pathway, NAGS plays an anaplerotic role and could therefore be subject to less stringent selection pressure, as long as NAG could be regenerated by transacetylation from other precursors.

We had initially expected that amino acids involved in binding of substrates would be conserved in all NAGS proteins, regardless of their biological role. An AcCoA binding motif (QXXGXG) in XcNAGS-K was identified based on its similarity with thialysine acetyltransferase (SSAT2), a member of the COG1246 family with known three-dimensional structure [45]. In SSAT2, binding of AcCoA is mediated mostly through hydrogen bonds with the main-chain oxygen and nitrogen atoms instead of by specific amino acid side chains [45]. Presumably the exact identity of these residues is less important as long as their side chains permit packing into the three-dimensional structure of either the COG1246 or DUF619 domains. This indirect form of amino acid interaction with substrates by the backbone instead of the side chains may explain, at least in part, the low conservation of NAGS motifs across phyla.

Present day NAGS from bacteria, plants, fungi and animals had been, until recently, considered either very distant relatives or not related at all [12]. This was probably the reason that mammalian NAGS was the last urea cycle gene to be identified and cloned [32]. The similarity of XcNAGS-K and mammalian NAGS, together with the wealth of genomic data has allowed us to begin to reconstruct evolutionary history of this gene. The bifunctional XcNAGS-K appears likely to be a direct descendant of the ancestral fusion protein that gave rise to extant mammalian NAGS and fungal NAGK. Purified recombinant XcNAGS-K was recently crystallized [52] and its structure and its close relationship with mammalian NAGS will provide new insights into structure and function of NAGS as well as genotype/phenotype correlations of NAGS deficiency.

## Conclusion

Based on the phylogenetic relationship and similar biochemical properties of XcNAGS-K and mammalian NAGS, we conclude that this bifunctional enzyme is closely related to the bacterial antecedent of mammalian NAGS.

## Methods

### Phylogenetic analysis

The GenBank database was queried with protein sequences of either human NAGS (hNAGS) or *argA *(NAGS) gene from *E. coli*. Truncated NAGS sequences and most of the putative NAGS genes were excluded from further analysis. Sequences of NAGS proteins from 28 species and four fungal NAGK proteins, listed in Additional file 2, were included in the phylogenetic analysis. These sequences were aligned using ClustalW alignment software with a Gonnet similarity matrix [53,54]. Conserved domains of vertebrate NAGS [24,34] were included in the alignment and subsequent phylogenetic analysis. Putative chloroplast and mitochondrial targeting signals were removed from plant and fungal NAGS sequences. To minimize gaps in the sequence alignment, additional blocks of amino acids from plant and fungal NAGS corresponding to extended loops were excluded from phylogenetic analysis based on their alignment with the *T. maritima *NAGK three-dimensional structure [41,42]. Blocks of amino acids that were excluded from the phylogenetic analysis are listed in the Additional file 2.

The PHYLIP 3.6 software package [55] was used for building phylogenetic trees. The protein distance matrix, based on either the Jones-Taylor-Thornton or PMB model of amino acid substitution [39,40], was calculated using the Protdist module. The neighbor module of the PHYLIP package was used to generate phylogenetic trees by the neighbor joining method. The Protpars module of the PHYLIP package was used to generate phylogenetic trees using the parsimony method. Bootstrap analysis was carried out with 1000 replicas for neighbor joining and parsimony trees.

### Identification and cloning of the *X. campestris argA-B *gene

The *X. campestris argA-B *gene was identified based on its similarity with mammalian NAGS. GenBank was queried with the human NAGS protein, and sequences annotated as *argB *from *X. campestris*, *X. axonopodis *and *X. fastidiosa *had slightly lower BLAST expectation scores than vertebrate NAGS genes. The *argB *gene from *X. campestris *was PCR amplified using genomic DNA (ATCC, catalog# 33913D) as the template and 5'-TCC**CATATG**TCCCTTCCTGCACAGCCCCAC-3' and 5'-AAC**GGATCC**TTATTATCACCCCAGCAAGGTGGGTTGACG-3' primers, with *Nde*I and *Bam*HI restriction sites shown in bold. The PCR product was cloned into a PCR^®^-Blunt II-TOPO^® ^plasmid using the Zero Blunt^® ^TOPO^® ^PCR cloning Kit (Invitrogen). The identity of 1345 bp insert was verified by DNA sequencing. This insert then was subcloned into a plasmid pET15b (Novagen) which allows overexpression of the recombinant protein (XcNAGS-K) with the N-terminal polyhistidine affinity tag and removal of the tag using thrombin protease. The resulting plasmid was termed pET15bXcNAGS-K.

### Overexpression and purification of XcNAGS-K

The pET15bXcNAGS-K plasmid was transformed into BL21(DE3) *E. coli *cells. Transformed cells were grown at 37°C and the overexpression of XcNAGS-K was induced with 0.2 mM isopropyl-β-D-thiogalactopyranoside at mid-log phase. Cells were harvested the following day by centrifugation at 3000 *g *for 15 min. at 4°C. The cell pellet was resuspended in Buffer A: 50 mM sodium phosphate pH 7.4 containing 300 mM NaCl, 10% glycerol and 10 mM β-mercaptoethanol (BME). Cells were disrupted by sonication for 10 min. on ice; the cell debris was removed by centrifugation at 15,000 *g *twice for 10 min. at 4°C. Nucleic acids were digested with RNaseA and DNaseI at 8 μg/ml each in lysates containing 5 mM MgCl_2 _for 30 min. at room temperature. The lysate was cleared by centrifugation at 16,000 *g *twice for 20 min. and 30 min. respectively.

Cleared lysate was loaded onto a HisTrap™HP Ni-affinity column (Amersham Biosciences) and pre-equilibrated with Buffer A. The column was washed with Buffer A containing 50 mM imidazole followed by elution of the bound XcNAGS-K with Buffer A containing 200 mM imidazole. Purified XcNAGS-K was dialyzed into 20 mM Tris-HCl buffer pH 7.4 containing 100 mM NaCl. The purified protein was stable in this buffer for two weeks at 4°C.

The N-terminal polyhistidine tag was removed from XcNAGS-K with thrombin protease. Thrombin was added to the purified protein at 1 U per 10 mg of XcNAGS-K, and the digestion was carried out for 24 hours at 4°C. The XcNAGS-K and thrombin were separated using Superdex 200™HR 10/30 gel-filtration column (Amersham Biosciences) equilibrated with 100 mM Tris-HCl buffer, pH 7.4, containing 100 mM NaCl and 5 mM BME. The XcNAGS-K without polyhistidine affinity tag is stable in this buffer for two weeks.

### Enzyme assays

Initial measurements of the synthase and kinase activities of XcNAGS-K were carried out as described previously [47,56]. In subsequent enzyme assays these methods were modified as follows. The synthase activity of XcNAGS-K was assayed in 100 mM Tris-HCl buffer, pH 9.0, 100 mM NaCl, 50 mM L-glutamate and 2 mM AcCoA in a total reaction volume of 100 μl. The assay was carried out for 5 min. at 30°C with 0.5 μg of enzyme in each reaction. The reaction was stopped with 100 μl of 30% TCA. NAG concentration was measured using liquid chromatography-mass spectroscopy (LC-MS) as described previously [32]. The K_m_^app ^and V_max _for glutamate and AcCoA were measured by varying glutamate concentration from 0 to 50 mM while keeping AcCoA at 2 mM and varying AcCoA concentration from 0 to 4 mM while keeping glutamate at 50 mM. The values of K_m_^app ^and V_max _were calculated using non-linear fitting to the Hill and Michaelis-Menten equations using the Gnuplot software package [57]. Where indicated, L-glutamate was replaced with 50 mM D-glutamate, L-glutamine or L-aspartate. The amounts of acetyl-D-glutamate, acetyl-L-glutamine or acetyl-L-aspartate formed were measured using LC-MS method, described above, with pure chemicals as standards. Inhibition of synthase activity was examined in the absence and the presence of 0.02, 0.05, 0.1, 0.2, 0.5, 1, 5, and 10 mM L-arginine

The NAG kinase activity of XcNAGS-K was measured using colorimetric assay, described previously [47], with some modifications. The enzymatic activity of 1.5 μg of XcNAGS-K was measured in 100 μl of 100 mM MES buffer, pH 6.0, 100 mM NaCl, 100 mM NAG, 20 mM ATP, 40 mM MgCl_2 _and 400 mM hydroxylamine. The reaction mixture was incubated at 30°C for 20 min. and the reaction was terminated with 100 μl of ferric chloride solution (5% FeCl_3_, 8% TCA and 0.3 M HCl). Absorbance of the colored product was measured at 540 nm. Where indicated, ATP was replaced with 20 mM CTP and UTP. Inhibition of the kinase activity was examined in the absence and presence of 0.02, 0.05, 0.1, 0.2, 0.5, 1, 10, 20 and 30 mM L-arginine.

The effect of pH on the synthase and kinase activities of XcNAGS-K was examined in a series of buffers: 100 mM acetate buffer (pH 4.5 and 5), 100 mM MES buffer (pH 5.5 and 6), 100 mM MOPS buffer (pH 6.5 and 7) and 100 mM Tris-HCl buffer (pH 7.5, 8, 8.5, 9 and 10). All buffers contained 100 mM NaCl. Substrate concentrations were 50 mM glutamate and 2 mM AcCoA in assays of synthase activity, and 100 mM NAG and 20 mM ATP in the kinase assays. The effects of different buffer components and ionic strength on the synthase activity of XcNAGS-K were examined by adding 100 mM MOPS, 100 mM acetate, 100 mM MES, 100 mM NaCl and 200 mM NaCl to enzymatic reaction carried out in Tris-HCl buffer. The effects of buffer components and ionic strength on the kinase activity of XcNAGS-K were examined by adding 100 mM MOPS, 100 mM acetate, 100 mM Tris-HCl, 100 mM NaCl and 200 mM NaCl to enzymatic reaction carried out in MES buffer.

The kinase activity was proportional to the concentration of purified XcNAGS-K and the rate of NAGP formation was linear with respect to time up to 20 min. The plots of dependence of the NAGP formation, with respect XcNAGS-K concentration and time, are provided in the Additional file 1.

## Authors' contributions

QQ carried out biochemical characterization of XcNAGS-K. HM participated in phylogenetic analysis and was involved in critical revisions of the manuscript. DS and MT have been involved in critical revisions of the manuscript. LC conceived the study, carried out phylogenetic analysis and drafted the manuscript. All authors read and approved the final manuscript.

## Supplementary Material

Additional file 1 **Catalysis of N-acetylglutamylphosphate formation by XcNAGS-K**. Graphs show rate of NAGP formation as a function of either XcNAGS-K concentration or time. The limitations of the colorimetric method for determination of kinase activity are explained in the text. Click here for file

Additional file 2 **Protein sequences that were included in phylogenetic analysis**. Protein sequences of NAGS and NAGK from 31 organisms (column A) were assembled for alignment and phylogenetic analysis. Accession numbers for each protein are shown in column B. Blocks of amino acids that were excluded from alignment to minimize gaps and the function of excluded blocks of residues are listed in columns C and D, respectively. Click here for file
