# Supplementary material for: A novel bifunctional N-acetylglutamate synthase-kinase from Xanthomonas campestris that is closely related to mammalian N-acetylglutamate synthase
Source: BMC Biochem. 2007 Apr 10;8:4. doi: 10.1186/1471-2091-8-4 (PMC1865377; doi:10.1186/1471-2091-8-4)
Supplement: Additional file 1 — Catalysis of N-acetylglutamylphosphate formation by XcNAGS-K. Graphs show rate of NAGP formation as a function of either XcNAGS-K concentration or time. The limitations of the colorimetric method for determination of kinase activity are explained in the text. [file 1471-2091-8-4-S1.doc]

**Catalysis of N-acetylglutamylphosphate formation by XcNAGS/K**

The rate of N-acetylglutamylphosphate (NAGP) formation was proportional to the concentration of purified XcNAGS/K (panel A) and linear with time (panel B).

A. B.

Our ability to measure slow rates of NAGP formation under these conditions was limited by the narrow dynamic range of the colorimetric assay for the kinase activity (ref). Increasing enzyme concentration and/or lengthening reaction time caused deviations from linearity. Possible reasons for this are inhibition by the products ADP and/or NAGP, changes in the oligomerization state of the enzyme and instability of the enzyme during longer incubation times.
